# Presence and Significance of Multiple Respiratory Viral Infections in Children Admitted to a Tertiary Pediatric Hospital in Italy

**DOI:** 10.3390/v16050750

**Published:** 2024-05-09

**Authors:** Velia Chiara Di Maio, Rossana Scutari, Lorena Forqué, Luna Colagrossi, Luana Coltella, Stefania Ranno, Giulia Linardos, Leonarda Gentile, Eugenia Galeno, Anna Chiara Vittucci, Mara Pisani, Sebastian Cristaldi, Alberto Villani, Massimiliano Raponi, Paola Bernaschi, Cristina Russo, Carlo Federico Perno

**Affiliations:** 1 Microbiology and Diagnostic Immunology Unit, Bambino Gesù Children’s Hospital, IRCCS, 00165 Rome, Italy; veliachiara.dimaio@opbg.net (V.C.D.M.);; 2 Multimodal Laboratory Research Unit, Bambino Gesù Children’s Hospital, IRCCS, 00165 Rome, Italy; rossana.scutari@opbg.net; 3 Hospital University Pediatrics Clinical Area, Bambino Gesù Children’s Hospital, IRCCS, 00165 Rome, Italysebastian.cristaldi@opbg.net (S.C.);; 4 Medical Direction, Bambino Gesù Children’s Hospital, IRCCS, 00165 Rome, Italy

**Keywords:** respiratory viruses, co-infection, multiplex PCR, children

## Abstract

Viral co-infections are frequently observed among children, but whether specific viral interactions enhance or diminish the severity of respiratory disease is still controversial. This study aimed to investigate the type of viral mono- and co-infections by also evaluating viral correlations in 3525 respiratory samples from 3525 pediatric in/outpatients screened by the Allplex Respiratory Panel Assays and with a Severe Acute Respiratory Syndrome-COronaVirus 2 (SARS-CoV-2) test available. Overall, viral co-infections were detected in 37.8% of patients and were more frequently observed in specimens from children with lower respiratory tract infections compared to those with upper respiratory tract infections (47.1% vs. 36.0%, *p* = 0.003). SARS-CoV-2 and influenza A were more commonly detected in mono-infections, whereas human bocavirus showed the highest co-infection rate (87.8% in co-infection). After analyzing viral pairings using Spearman’s correlation test, it was noted that SARS-CoV-2 was negatively associated with all other respiratory viruses, whereas a markedly significant positive correlation (*p* < 0.001) was observed for five viral pairings (involving adenovirus/human bocavirus/human enterovirus/metapneumoviruses/rhinovirus). The correlation between co-infection and clinical outcome may be linked to the type of virus(es) involved in the co-infection rather than simple co-presence. Further studies dedicated to this important point are needed, since it has obvious implications from a diagnostic and clinical point of view.

## 1. Introduction

Acute respiratory infections (ARIs) are the leading cause of infections in pediatric populations worldwide and are one of the most frequent causes of consultation or admission to healthcare facilities in pediatric services [1]. ARIs are classified as upper and lower respiratory tract infections, based on the respiratory tract involved. Clinically, upper respiratory tract infections are usually characterized by mild symptoms and are generally self-limiting, whereas lower respiratory tract infections may cause severe clinical manifestations (such as bronchitis, bronchiolitis, and/or pneumonia) [2]. A variety of respiratory viruses are involved in the etiology of respiratory tract infections in pediatric populations, including adenovirus (ADV), human bocavirus (HBoV), human enterovirus (HeV), human rhinoviruses (HRV), respiratory syncytial viruses (RSV A and B), metapneumoviruses (MPV), four parainfluenza viruses (PIVs 1–4), influenza viruses (Flu A and B), and human coronaviruses (HCoVs). Some of these viruses, such as RSV or Flu A and B, typically show extensive seasonal variation in prevalence, whereas others are commonly present throughout all seasons [3]. In the last two years, with the introduction of control measures due to the Severe Acute Respiratory Syndrome COronaVirus 2 (SARS-CoV-2) pandemic, the epidemic seasonality of other respiratory viruses changed [4,5,6,7]. However, the mitigation measures after the pandemic allowed these viruses, including SARS-CoV-2, to begin circulating once again [8]. Currently, multiplex polymerase chain reaction (PCR) panel assays, which allow for the simultaneous detection of different pathogens in a specimen with high sensitivity and short turnaround time, are becoming more commonly used in diagnostics [9]. Thanks to the introduction of these molecular assays also in the diagnosis of respiratory tract infections, it is now possible to better study and understand the epidemiology and pathogenesis of respiratory viruses. Since various respiratory viruses may be circulating in the same period, they can infect the same respiratory tract either concurrently or sequentially [10]. As a result, there are three possible major effects of these interactions: viral synergy, viral interference, and viral non-interference [11]. In fact, infection by a first virus can have a synergistic interaction or an antagonistic mechanism, thus promoting or reducing the infection and replication of another virus, by several pathophysiological factors, including synergistic damage to the epithelium or, by contrast, to the production of interferon [10]. If the presence of the first virus has no effect on the replication of another, this is defined a non-interference [11]. Viral co-infections in pediatric populations with ARIs are frequently reported by multiplex PCR, particularly in those who are hospitalized [12,13,14,15]. The impact of viral co-infection and its correlation with the disease severity of respiratory tract infection in the pediatric population remains unclear. In some studies, viral co-infection was associated with disease progression and the worsening of symptoms [12,14]. In contrast, no differences were observed by other authors in the disease outcome compared to viral mono-infection [16,17]. This variability can be driven by various factors, including the number of patients under studies (often limited) and the lack of correlation among specific viruses. Convincing evidence in this setting has obvious diagnostic and therapeutic implications. In general, virus–virus interactions and their occurrence at the population level are still poorly understood and need to be thoroughly investigated, especially after the SARS-CoV-2 pandemic. Indeed, as shown above, whether specific viral interactions enhance or diminish the severity of respiratory disease is still controversial. Therefore, starting from these different observations, the preliminary aim of the study was to describe the presence of viruses in the respiratory specimens collected from a large cohort of children and adolescents (0–18 years old) admitted to a large tertiary pediatric hospital in Rome between 1 January 2022 and 30 June 2023. Then, our main aim was to investigate the distribution and type of viral mono- and co-infections in respiratory samples included in the analysis. In a subgroup of pediatric patients whose ARI (upper or lower) diagnosis was available, we also evaluated the type of viral detections.

## 2. Materials and Methods

### 2.1. Sample Characteristics and Inclusion Criteria of the Analysis

Between 1 January 2022 and 30 June 2023, 10,018 respiratory samples from 5171 children and adolescents (0–18 years, both inpatients and outpatients) with clinically relevant respiratory symptoms were tested using the Allplex Respiratory Panel Assays (Seegene, Seoul, Republic of Korea) at the Bambino Gesù Children’s Hospital in Rome, Italy. If a patient had multiple respiratory samples, we selected the first one carried out. In most cases, this was the one performed at the time of hospital admission (inpatient and outpatient). Respiratory samples belonging to the same patient with a temporal distance >30 days were considered as a new case. Data from 3525 pediatric patients with inclusion criteria were integrated with SARS-CoV-2 test results obtained by either molecular or antigenic methods if the test was performed within 72 h before or after the multiplex respiratory panel test (Figure S1). Clinical information regarding the type of ARI (upper or lower respiratory tract infections) diagnosed at the time of hospital admission was collected for a large subgroup of respiratory samples from 1011 pediatric patients (Figure S1).

### 2.2. Allplex Respiratory Panel

The Allplex Respiratory Panel Assay is a closed multiplex PCR system for the simultaneous detection of virus respiratory pathogens that includes extraction, amplification, detection, and analysis of samples. All steps, from nucleic acid extraction to final pathogen detection, were carried out automatically. Nucleic acid extraction and PCR preparation were performed in a one-step process using Hamilton (Seegene Inc., Seoul, Republic of Korea). A multiplex PCR assay for 16 different respiratory tract viruses was performed in three different PCR multiplex panels. These panels allow for the identification of 16 different viruses: Flu A and B, RSV A and B, ADV, HeV, PIV1-4, MPV, HBoV, HRV, and three human coronaviruses (CoV NL63/229E/OC43). Each reaction mixture contained 8 µL of extracted nucleic acid and 17 µL of one-step RT-PCR master mix (5 µL of RP MOM, 5 µL of RNase-free water, 5 µL of 5× real-time one-step buffer, and 2 µL of real-time one-step enzyme) at a final volume of 25 µL. Multiplex RT-PCR was performed using a CFX96™ Real-time PCR System (Bio-Rad Laboratories, Hercules, CA, USA). The results were analyzed automatically using Seegene Viewer V3.0 (Seegene Inc., Seoul, Republic of Korea) and interpreted according to the manufacturer’s instructions. Following the datasheet indications for interpretation of the results, the sample was considered positive for the presence of the pathogen if the Cycle threshold (Ct) value was ≤42 cycles. A negative specimen was defined by the absence of amplification for any pathogen with the presence of amplification of the internal control.

### 2.3. SARS-CoV-2 Tests

Data of multiplex respiratory panel tests were integrated with SARS-CoV-2 test results assessed by antigenic or molecular tests. SARS-CoV-2 antigen testing was performed using the electrochemiluminescence immunoassay (ECLIA) Roche Elecsys SARS-CoV-2 Antigen on a Roche Cobas e411 (Roche Diagnostics GmbH, Mannheim, Germany) or the fluorescent immunoassay RADT STANDARD F COVID-19 Ag FIA on an F2400 analyzer (SD BIOSENSOR, Korea). According to the manufacturers’ instructions, a result of cut-off index (COI) ≥ 1.0 was interpreted as reactive for SARS-CoV-2 antigen. Molecular tests were performed for the detection of the SARS-CoV-2 virus through reverse transcription (RT), followed by real-time PCR from RNA extracted from respiratory samples by different assays according to the manufacturers’ instructions. The Cepheid Xpert Xpress CoV-2 plus performed on Cepheid’s GeneXpert^®^DX system (Cepheid, Sunnyvale, CA, USA) is an assay that amplifies and detects unique sequences in the nucleocapsid (N), envelope (E), and RNA-dependent RNA polymerase (RdRP) genes of the SARS-CoV-2 genome. The SARS-CoV-2 ELITe MGB Kit^®^ (Elitechgroup, Turin, Italy), performed using the ELITe InGenius instrument (Elitechgroup, Turin, Italy), is an assay that amplifies and detects unique sequences in the RdRp and ORF8 genes of the SARS-CoV-2 genome. 

### 2.4. Interpretation of Data and Statistical Analysis

Based on the results obtained, samples were classified as positive or negative for each pathogen. Among the positive results, the percentage of samples with mono-infection and co-infection was determined based on the number of pathogens identified. Co-infection was defined as positive detection of >1 virus in the same sample. Descriptive statistics are expressed as median values and interquartile range (IQR) for continuous data and number (percentage) for categorical data. Statistical comparisons were performed using Fisher’s exact and Mann–Whitney tests for categorical and continuous variables, respectively. A value of *p* less than 0.05 was considered statistically significant. The Spearman’s rank correlation coefficient was calculated to evaluate correlations between respiratory viruses. Data were analyzed using statistical software package SPSS (v32.0; SPSS Inc., Chicago, IL, USA).

## 3. Results

### 3.1. Seasonal Distribution of Non-SARS-CoV-2 Respiratory Viruses in Samples Analyzed 

During the study period, a total of 10,018 respiratory samples from 5171 children and adolescents with a median age of 3.0 years (interquartile range [IQR]: 0.5–6.9) were screened for respiratory viruses at the Bambino Gesù Children’s Hospital in Rome, Italy. Of these, 6061 (60.5%) were positive for at least one virus (Figure S1). For some viruses, a different seasonal pattern was observed during 2022 and the first part of 2023. As expected, RSV, Flu A and B were mainly detected in the cold months, but with some differences during the two years (Figure S2). Considering RSV, the detection of this virus was highest from November to February 2022. During the cold months of January–February 2022, RSV A was more prevalent than RSV B (20.8% vs. 7.8%, *p* < 0.001). In contrast, between November 2022 and January 2023, RSV B circulated predominantly compared to RSV A (57.9% vs. 10.9%, *p* < 0.001). A different prevalence was also observed for Flu A and Flu B during the cold months of 2022 and 2023. In particular, the prevalence of Flu A was the highest from November 2022 to January 2023. By comparing January–February 2022 and January–February 2023, a significant increase in Flu A prevalence was observed (0.7% vs. 15.7%, *p* < 0.001). Circulation of Flu B was rarely observed during 2022 (below 1%), whereas the prevalence of this virus increased significantly in 2023, with the highest detection in March 2023 (3.6%, N = 23). Other viruses like ADV, HBoV, HeV, and HRV were detected throughout the 2022 year, with no significant fluctuation in prevalence during the different months, independent of the cold temperatures (Figure S2). A similar trend was also observed during the winter and spring seasons of 2023 (Figure S2). 

### 3.2. General Results of Samples Included in the Analysis

Of the 10,018 respiratory specimens initially considered, 3525 of them, each taken from 3525 patients, met the inclusion criteria, i.e., multiplex respiratory panel test and a SARS-CoV-2 test performed within 72 h available (see Section 2.1 and Figure S1). Of them, 1881 (53.4%) were from males, and 1644 (46.6%) were from from females. The median age was 3.3 years (IQR: 2.7–4.9). Samples were also stratified according to Eunice Kennedy Shriver National Institute of Child Health and Human Development age stage terminology [18]. By stratifying samples based on these categorical ages, most of them were from early childhood (2.1 to 5 years of age; N = 1028, 29.2%) and infants (29 days to 12 months; N = 770, 21.8%), followed by middle childhood (6 to 11 years of age; N = 579, 16.4%), toddlers (13 months to 2 years; N = 405, 11.5%), neonates (birth to 28 days; N = 377, 10.7%), and adolescents (12 to 18 years of age; N = 366, 10.4%). The large majority of respiratory specimens was collected by a nasopharyngeal aspirate (N = 2406, 68.3%) or nasopharyngeal swab (N = 1089, 30.9%; see Table 1).

### 3.3. Detection of Respiratory Viruses

**Figure 1:**
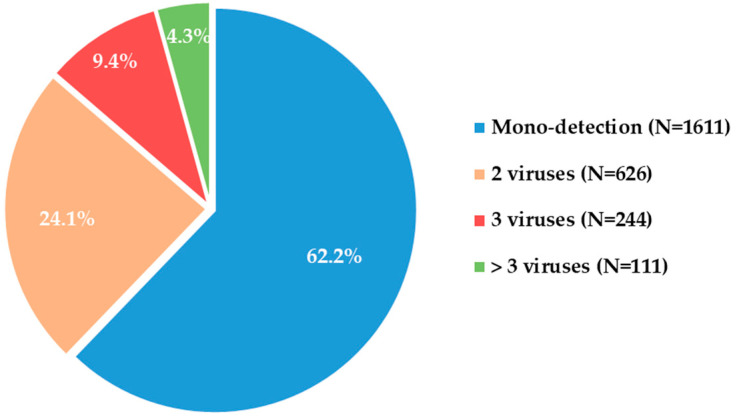
Distribution of viral detections in positive respiratory samples with a SARS-CoV-2 test available (N = 2592).

Of the 3525 respiratory samples analyzed from the corresponding 3.525 patients, 73.5% (N = 2592) were positive for at least one virus. Viral mono-infection was found in 62.2% (N = 1611) of positive samples, and co-infection was observed in 37.8% (N = 1981) of the specimens. Often, co-infection involved two viruses (24.1%, N = 626), and the detection of >3 viruses was less frequent but still remarkable (9.4%, N = 244 for 3, and 4.3%, N = 111 for >3 viruses, Figure 1). 

A different distribution of viral mono- and co-infections was observed among age groups. Indeed, viral co-infection was significantly higher in samples from toddlers and early childhood (55.0%, N = 183 and 46.0%, N = 383, respectively) compared to samples from adolescents and neonates (18.4%, N = 155 and 22.4%, N = 210, respectively) (*p* < 0.001) (Figure S3). 

### 3.4. Distribution of Detected Viruses and Viral Pair Relations

Overall, HRV was detected in 48.5% of all samples, followed by ADV (16.5%), SARS-CoV-2 (16.0%), HBoV (12.1%), and RSV B (11.9%) (Table 2). 

By analyzing the distribution of each virus in mono- or co-infection, substantial differences were observed. The majority of the viruses were more often detected as a part of a co-infection: the highest co-infection rate was observed for HBoV (87.8% co-infection vs. 12.2% mono-infection, *p* < 0.001) and HeV (80.5% co-infection vs. 19.5% mono-infection, *p* < 0.001) (Table 2). Considering viral co-infections, viral pairings frequently detected mainly involved HRV with other viruses, thus reflecting the high number of this virus detection in the overall samples analyzed (Table 2 and Table S1). In particular, HRV was observed more often in co-infection (55.0% co-infection vs. 45.0% mono-infection, *p* < 0.001). In contrast, Flu A was mainly seen in mono-infection (55.1% mono-infection vs. 44.9% co-infection, *p* = 0.052) (Table 2 and Table S1). SARS-CoV-2 was also mainly seen in mono-infection (63.3%, N = 262 mono-infection vs. 36.7%, N = 152 co-infection, *p* = 0.619). Moreover, a pairwaise analysis between SARS-CoV-2 and other viruses was performed. This analysis highlighted a statistically significant distribution of this virus mainly in mono-infection (*p* < 0.05).

### 3.5. Correlations between Respiratory Viruses

Correlations between respiratory viruses were also evaluated with Spearman’s rank correlation test. The data showed 13 positive correlations and 19 negative correlations that were statistically significant among the viral pairs identified (Table 3). In particular, a markedly significant correlation (*p* < 0.001) was observed for five positive associations and for four negative associations. Significantly positive correlations were found between ADV and HBoV (r = 0.106, *p* < 0.001), ADV and HeV (r = 0.094, *p* < 0.001), HBoV and HeV (r = 0.085, *p* < 0.001), HBoV and MPV (r = 0.081, *p* < 0.001), and HeV and HRV (r = 0.110, *p* < 0.001) (Table 3). In contrast, significantly negative correlations were observed between OC43 and HRV (r = −0.077, *p* < 0.001), Flu A and HRV (r = −0.0164, *p* < 0.001), HRV and RSV A (r = −0.091, *p* < 0.001), and HRV and RSV B (r = −0.127, *p* < 0.001) (Table 3). In addition, SARS-CoV-2 was negatively associated with all other respiratory viruses tested (*p* < 0.05), thus confirming the high prevalence of this virus in mono-detection. Thus, some viruses had a significant association (either negative or positive) with other viruses, confirming that their co-presence is not casual, but is regulated by complex factors that can include cellular target(s), replication capacity, pathogenetic characteristics, seasonality, etc.

### 3.6. Analysis of Viral Detection in Samples of Patients with ARI Diagnosis 

The next step of the work was to evaluate viral distribution in a subset of 1011 samples, each belonging to pediatric patients with a defined diagnosis of ARI (upper or lower) at the time of hospital admission. Among the 1011 samples, 378 (37.0%) were from an upper ARI, and 633 (63.0%) were from from a lower ARI. We evaluated the correlation of mono-coinfection with the epidemiological and clinical data. Looking at demographic characteristics, no statistically significant differences were observed between groups, except for a higher prevalence of early childhood and longer duration of hospitalization in the group with lower ARI (*p*: 0.012 and <0.001, respectively) (Table 4). 

**Figure 2:**
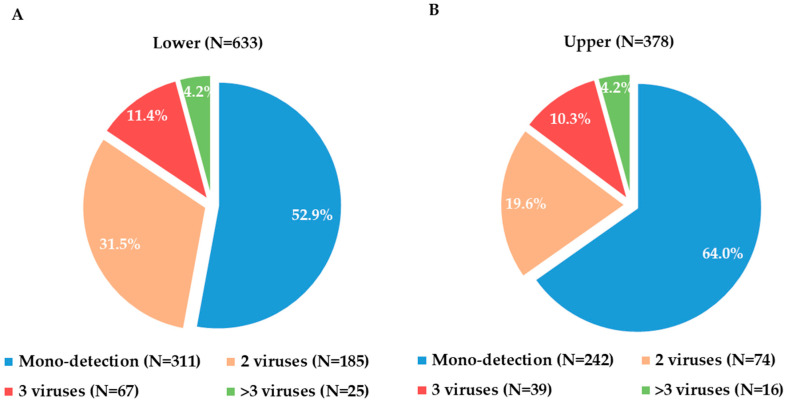
Distribution of viral detections in lower (panel **A**) and upper (panel **B**) acute respiratory infections (ARIs).

By analyzing the distribution of viral detection in the two groups, a higher prevalence of co-infection was observed in the lower respiratory tract compared to the upper respiratory tract infections (47.1% vs. 36.0%, *p* = 0.003) (Figure 2). Overall, SARS-CoV-2 was the virus mainly detected in upper ARI, mostly (see above) in mono-infection (66.1% in mono-infection vs. 33.9% in co-infection, *p* = 0.390). RSV B and (not totally unexpected, see the Discussion section) HRV were the most frequently detected viruses in lower ARI; both were mainly involved in co-infections (HRV in particular: 69.8% in co-infection vs. 30.2% in mono-infection, *p* < 0.001, whereas RSV B was found 52.6% of the time in co-infection vs. 47.4% of the time in mono-infection, *p* = 0.07).

## 4. Discussion

Two years after of the coronavirus disease 2019 (COVID-19) pandemic, and as a result of the relaxation of the mitigation measures, there is now a co-circulation of SARS-CoV-2 with other respiratory viruses. In this new epidemiological scenario, the role of viral interactions due to co-infection is yet to be thoroughly investigated. Respiratory viruses may infect various cells within the respiratory tract, including ciliated epithelial cells, alveolar cells, and immune cells. The co-presence of different respiratory viruses in the same respiratory tract can lead to specific viral interactions that depend on several aspects, including host factors, the seasonality of viruses, and their cell tropism. Indeed, if two viruses infect the same cell, there is a competition mechanism in which one virus can inhibit the replication of the other. Instead, if two viruses infect different cell types, they are more likely to be co-present. What is driving these positive and negative interactions during co-infections is unknown, but studying viral correlations at the population level can help to provide insight into these mechanisms and the possible role in the disease severity. This may be particularly important for the pediatric population, as viral co-infections are frequently observed among children compared to adults [19]. For this reason, this study investigated as a primary objective the distribution and type of viral mono- and co-infections by also evaluating the type of viral correlations in a large number of respiratory samples collected from children and adolescents admitted to the Bambino Gesù Children’s Hospital in Rome between 1 January 2022 and 30 June 2023. A preliminary epidemiological analysis was also performed to evaluate the distribution of respiratory viruses other than SARS-CoV-2. 

By analyzing the overall dataset of 10,018 respiratory samples (Figure S1), collected in a post-pandemic period, a substantial re-circulation of respiratory viruses other than SARS-CoV-2 was observed. A variation in seasonal pattern was observed particularly for RSV and influenza viruses in the 2022–2023 season, which marked the return of the circulation of these viruses at almost pre-pandemic levels, as also observed in other studies [20,21,22,23]. In this setting, considering the principal aim of this study, we evaluated the number and type of viral detection in a subgroup of 3525 patients screened for respiratory viruses, also considering SARS-CoV-2. Overall, the majority of positive samples (62.2%) were characterized by the presence of a single virus. Co-infection was found in 37.8% of respiratory specimens analyzed, particularly in samples from toddlers (13 months to 2 years) and early childhood (2 to 5 years of age). This result is in line with recent studies, which found that the age group with the highest viral co-infection rate is, in general, that of children aged 0–5 years old [4,24,25]. By analyzing the distribution of each virus in mono- or co-infection, the data show that the highest co-infection rate was observed for HBoV (87.8% in co-infection). This virus was also involved in some significantly positive correlations with ADV, MPV, and HeV. HBoV is usually characterized by prolonged viral shedding, with a persistence of its DNA described for up to 3 months in outpatients and up to 1 year in hospitalized children after acute infection [26]. This can in part explain the high frequency of HBoV co-detection with other respiratory viruses [26,27]. In this study, a single sample per patient was analyzed, and in most cases, this coincided with that performed at hospital admission. For patients with an HBoV-positive respiratory test, we assessed whether longitudinal samples were also present, and indeed, a persistence of HBoV up to 4 months was observed.

The phenomenon described above raises the question as to whether HBoV has a causative pathogenetic role for co-infection with other viruses, or whether it is found only as a co-detected virus, since it can persist in the host for a long time. Recent studies reported that low HBoV viral loads were detected more often in co-infection [26,28]. In contrast, high HBoV loads were observed mainly in the absence of other respiratory viruses, suggesting a causative role of this virus in the etiology of the respiratory disease [26,28]. By analyzing the threshold cycles (Ct) of HBoV-positive samples, we also observed a trend toward lower CT values (that is, high viral load) when HBoV was detected in mono-infection versus when it was detected in co-infection, though the difference was not statistically significant. Therefore, in the study of viral co-infection, viral load may serve also as an important value to understand which virus may have a greater influence on the clinical severity of the respiratory disease. This aspect became particularly relevant during the COVID-19 pandemic. Indeed, Ct values can be used to estimate the viral load of SARS-CoV-2 as a marker of infectivity and to identify patients at higher risk for morbidity or severe outcome [29].

In our study, SARS-CoV-2 and Flu A tend to be found mostly in mono-infection. Interestingly; another recently published study reports that in children, most viral co-infections were found at significantly reduced rates relative to that expected from the incidence of each virus, especially those involving SARS-CoV-2 and influenza [30].

Another aspect analyzed in this study included the interactions between respiratory viruses through analysis of correlation. Significantly negative associations were observed particularly for SARS-CoV-2 with all other respiratory viruses tested. Moreover, a negative correlation was also observed for HRV with RSV A and RSV B and for Flu A with HRV. The biological mechanism related to viral interference may in part help to explain the negative correlations observed in this study. Indeed, recent findings reported that SARS-CoV-2 replication can be impaired by a primary HRV infection [31]. It has also been found that SARS-CoV-2 is particularly susceptible to negative interference by Flu and RSV, i.e., viruses that trigger a strong IFN response [32]. It has also been well demonstrated that a previous infection with rhinovirus inhibits infection with influenza A virus, by activating antiviral defenses in the target tissue against both viruses [33]. A negative association between HRV and RSV was also reported in different epidemiological studies including large pediatric populations [34]. In fact, it has been shown that children with prior exposure to RSV had a lower incidence of HRV than those without a previous RSV infection. Other factors unrelated to viral interference could also contribute to the negative correlations between specific viruses, such as differences in virus seasonality based on environmental factors or differences in virus host range (e.g., viruses that preferentially infect different age groups). Further longitudinal studies in a large cohort of pediatric patients are needed to clarify this point.

Evidence concerning the severity of disease in the presence of a viral co-infections compared to single viral infections is conflicting. Indeed, some studies have reported an increased risk for lower respiratory tract infections in hospitalized children [15,35], whereas others were unable to identify clinical differences [36,37]. In our study, no analysis related to disease severity was performed. However, we investigated the type of viral detections in a subgroup of pediatric patients whose ARI (upper or lower) diagnosis was available. We observed a higher prevalence of viral co-infections particularly in samples from patients with a lower respiratory tract infection compared to patients with an upper respiratory tract infection. The viruses that mainly characterized viral mono- and co-infection in lower respiratory tract infection were HRV and RSV B. These data are concordant with the evidence showing that HRV may also be associated with lower respiratory tract infections alongside RSV in infants/children [38]. Moreover, despite the negative interaction between RSV and HRV, when co-infection is present, an increase in disease severity is reported, as compared to a single RSV infection [34].

One limitation of this study is that it did not also consider the presence of potential bacterial coinfections. Another limitation is that it was not possible to retrieve information on the patients’ vaccination status (i.e., influenza vaccine). Vaccination can impact viral dynamics and kinetics. Vaccinated individuals may have a shorter infectivity duration and a lower viral load. Therefore, this can potentially impact viral interactions in coinfection. This aspect is still poorly investigated and deserves further investigation.

Taken together, all our data suggest that the correlation between co-infection and clinical outcome may be linked to the type of virus(es) involved in the co-infection rather than a simple co-presence. Further studies dedicated to this important point are needed, since it has obvious implications from a diagnostic and clinical point of view (i.e., identification of patients that may have a worse clinical outcome when carrying a specific association of viruses rather than a general mono- vs. multiple infection). Lastly, by considering the upper respiratory tract infection, SARS-CoV-2 was the most frequently detected pathogen. Currently, there is a global dominance of the Omicron variant [39]. Compared with previous variants, it has shown greater tropism for the upper respiratory tract and/or bronchial tissue [40,41,42]. This change in tropism can help to explain the greater concentration of the virus in the upper respiratory tract, which makes its detection in this respiratory tract more likely.

## 5. Conclusions

In conclusion, in this study, the high number of respiratory samples from pediatric patients analyzed highlighted different patterns of positive and negative correlations between viral pairings. In particular, SARS-CoV-2 was negatively associated with all other respiratory viruses, whereas a markedly significant positive correlation was observed for five viral pairings (involving adenovirus/human bocavirus/human enterovirus/metapneumoviruses/rhinovirus). Viral co-infection was frequently observed in samples from children with a lower respiratory tract infection compared to those with an upper respiratory tract infection, thus suggesting a role for co-infections in the prognosis of these patients. Further studies are required to confirm the significance of the viral pairings observed in this analysis and to elucidate the virologic mechanisms underlying the association that may lead to cooperation or competition between co-detected viruses. Clinical data will help to elucidate the role of multiple viral co-infections on the severity of respiratory disease.

## Figures and Tables

**Table 1:** General characteristics of 3525 patients included in the analysis.

Total, N	3525
Demographic characteristics	
Male, N (%)	1881 (53.4)
Female, N (%)	1644 (46.6)
Age, years, median (IQR)	3.3 (2.7–4.9)
Age category	
Neonates (birth–28 days), N (%)	377 (10.7)
Infant (29 days–12 months), N (%)	770 (21.8)
Toddler (13 months–2 years), N (%)	405 (11.5)
Early childhood (2.1–5 years), N (%)	1028 (29.2)
Middle childhood (6–11 years), N (%)	579 (16.4)
Adolescence (12–18 years), N (%)	366 (10.4)
Sample type	
Nasopharyngeal aspirate, N (%)	2406 (68.3)
Nasopharyngeal swab, N (%)	1089 (30.9)
Bronchoalveolar lavage, N (%)	30 (0.85)

IQR, interquartile range.

**Table 2:** Absolute number and percentage of positive samples for 17 different viruses as mono-detection or co-detection (2 viruses or >2 viruses).

	AdV (N = 428)	HBoV (N = 314)	HCoV-229A (N = 36)	HCoV-NL63 (N = 45)	HCoV-OC43 (N = 132)	HeV (N = 278)	Flu A (N = 174)	Flu B (N = 36)	MPV (N = 234)	PIV1 (N = 54)	PIV2 (N = 64)	PIV3 (N = 193)	PIV4 (N = 40)	HRV (N = 1256)	RSV A (N = 79)	RSV B (N = 308)	SARS-CoV-2 (N = 414)
1 Virus (N = 1611)	122 (28.5)	38 (12.0)	14 (38.9)	13 (28.9)	37 (28.0)	54 (19.4)	96 (55.1)	19 (52.8)	81 (34.6)	14 (25.9)	30 (46.9)	80 (41.4)	14 (35.0)	566 (45.1)	36 (45.6)	135 (43.8)	262 (63.3)
2 Viruses (N = 626)	140 (32.7)	105 (33.4)	13 (36.1)	16 (35.5)	43 (32.6)	79 (28.4)	46 (26.4)	9 (25.0)	77 (32.9)	15 (27.8)	17 (26.6)	59 (30.6)	20 (50.0)	399 (31.8)	26 (32.9)	99 (32.1)	89 (21.5)
>2 Viruses (N = 355)	166 (38.8)	171 (54.4)	9 (25.0)	16 (35.5)	52 (39.4)	145 (52.1)	32 (18.4)	8 (22.2)	76 (32.5)	25 (46.3)	17 (26.6)	54 (27.8)	6 (15.0)	291 (23.2)	17 (21.5)	74 (24.0)	63 (15.2)
*p*-value *	<0.001	<0.001	0.005	<0.001	<0.001	<0.001	0.052	0.299	<0.001	<0.001	0.013	<0.001	0.001	<0.001	0.003	<0.001	0.619

* Two-sided *p*-values were calculated by Fisher’s exact test.

**Table 3:** Correlations among respiratory viruses.

Virus 1	Virus 2	Spearman’s Rho	*p*-Value
Positive correlations			
ADV	HBoV	0.106	<0.001
ADV	HeV	0.094	<0.001
HBoV	HeV	0.085	<0.001
HBoV	MPV	0.081	<0.001
HeV	HRV	0.110	<0.001
ADV	NL63	0.044	0.024
HBoV	HRV	0.068	0.001
HBoV	OC43	0.048	0.014
HBoV	PIV1	0.045	0.021
OC43	RSVA	0.061	0.002
OC43	RSVB	0.045	0.022
HeV	MPV	0.056	0.004
HeV	PIV1	0.045	0.021
Negative correlations			
OC43	HRV	−0.077	<0.001
Flu A	HRV	−0.164	<0.001
HRV	RSV A	−0.091	<0.001
HRV	RSV B	−0.127	<0.001
ADV	Flu A	−0.061	0.002
ADV	RSV B	−0.067	0.001
ADV	RSV A	−0.055	0.005
HEV	RSV B	−0.050	0.011
HEV	Flu A	−0.048	0.014
MPV	HRV	−0.066	0.001
MPV	RSV A	−0.048	0.014
MPV	PIV2	−0.041	0.035
PIV 3	RSV B	−0.059	0.003
PIV 3	HRV	−0.054	0.006
Flu A	PIV3	−0.047	0.017
Flu A	MPV	−0.041	0.035
229E	HEV	−0.041	0.036
229E	HRV	−0.043	0.030
PIV 2	RSV B	−0.043	0.026

**Table 4:** General characteristics of samples belonging to pediatric patients with a diagnosis of acute respiratory infection (ARI, upper or lower) at the time of hospital admission.

	Total ARI (N = 1011)	Upper ARI (N = 378)	Lower ARI (N = 633)	*p*-Value *
Male, N (%)	553 (54.5)	195 (51.6)	358 (56.5)	0.133
Age, years, median (IQR)	0.9 (0.2–3.6)	0.8 (0.1–3.1)	1.0 (3.0–7.9)	0.451
Neonates, N (%)	177 (17.5)	69 (18.2)	108 (17.0)	0.669
Infant, N (%)	351 (34.7)	135 (35.7)	216 (34.1)	0.633
Toddler, N (%)	112 (11.1)	49 (13.0)	63 (9.9)	0.148
Early childhood, N (%)	225 (22.2)	68 (18.0)	157 (24.8)	0.012
Middle childhood, N (%)	89 (8.8)	39 (10.3)	50 (7.9)	0.207
Adolescence, N (%)	57 (5.6)	18 (4.8)	39 (6.3)	0.399
Length of hospitalization (days, median IQR)	4.3 (2.9–7.1)	3.6 (2.7–5.5)	5.0 (3.0–7.9)	<0.001
Clinical manifestation				
Bronchitis	178 (17.6)	-	178 (28.1)	-
Bronchiolitis	257 (25.4)	-	257 (40.6)	-
Pneumonia	198 (19.6)	-	198 (31.3)	-

IQR, interquartile range; * two-sided *p*-values were calculated by Fisher’s exact test or Mann–Whitney test, as appropriate.

## Data Availability

Data are contained within the article (and its Supplementary Materials).

## Supplementary Materials

The following supporting information can be downloaded at https://www.mdpi.com/article/10.3390/v16050750/s1: Figure S1. Sample characteristics and inclusion criteria of the analysis; Figure S2. Distribution of community respiratory viruses from January 2022 to June 2023; Figure S3. Distribution and prevalence of viral mono- and co-detection within age groups; Table S1. Matrix table showing viral pairings observed in the samples analyzed.
