# Supplementary material for: Presence and Significance of Multiple Respiratory Viral Infections in Children Admitted to a Tertiary Pediatric Hospital in Italy
Source: Viruses. 2024 May 9;16(5):750. doi: 10.3390/v16050750 (PMC11126044; doi:10.3390/v16050750)
Supplement: Supplementary file 1 [file viruses-16-00750-s001.zip › Supplementary Table S1.pdf]

Supplementary Table S1. Matrix table showing viral pairings observed in the samples analysed

Co-detected virus	AdV	HBoV	229E	NL63	OC43	HEV	Flu A	Flu B	MPV	PIV1	PIV2	PIV3	PIV4	HRV	RSV A	RSV B	SARS-CoV-2	Total
AdV		85	2	13	22	74	14	2	32	7	5	26	3	205	4	30	29	428
HBoV	85		6	5	25	56	14	4	48	12	6	23	4	181	8	36	37	314
229E	2	6		1	2	0	1	0	5	0	0	3	0	11	2	2	2	36
NL63	13	5	1		2	4	0	0	5	0	0	3	0	21	0	3	0	45
OC43	22	25	2	2		13	14	1	16	1	0	7	1	42	10	24	4	132
HEV	74	56	0	4	13		9	4	38	11	10	26	3	179	4	20	21	278
Flu A	14	14	1	0	14	9		1	8	4	1	5	0	31	4	16	5	174
Flu B	2	4	0	0	1	4	1		3	2	0	0	0	12	0	2	0	36
MPV	32	48	5	5	16	38	8	3		6	1	10	1	89	1	19	16	234
PIV1	7	12	0	0	1	11	4	2	6		3	3	0	33	1	8	1	54
PIV2	5	6	0	0	0	10	1	0	1	3		1	0	29	0	2	7	64
PIV3	26	23	3	3	7	26	5	0	10	3	1		0	75	2	10	13	193
PIV4	3	4	0	0	1	3	0	0	1	0	0	0		19	0	2	1	40
HRV	205	181	11	21	42	179	31	12	89	33	29	75	19		18	96	85	1256
RSV A	4	8	2	0	10	4	4	0	1	1	0	2	0	18		8	7	79
RSV B	30	36	2	3	24	20	16	2	19	8	2	10	2	96	8		13	308
SARS-CoV-2	29	37	2	0	4	21	5	0	16	1	7	13	1	85	7	13		414

The total number (N) represents the sum of mono- and co-detection for each virus. In the white boxes for each viral parings are reported the total of co-detection observed as 2,3 or >3 associations.

ADV: Adenovirus, HBoV: Human bocavirus, HCoV-229E: Human coronavirus 229E, HCoV-OC43: Human coronavirus OC43, HeV: Human enterovirus, FluA: Influenza A virus, FluB: Influenza B virus; MPV: Human metapneumovirus, PIV1: Human parainfluenza virus 1, PIV2: Human parainfluenza virus 2, PIV3: Human parainfluenza virus 3, PIV4: Human parainfluenza virus 4, HRV: Human rhinovirus, RSVA: Respiratory syncytial virus A, RSVB: Respiratory syncytial virus B, SARS-CoV-2: Severe acute respiratory syndrome coronavirus.
