# Supplementary material for: Presence and Significance of Multiple Respiratory Viral Infections in Children Admitted to a Tertiary Pediatric Hospital in Italy
Source: Viruses. 2024 May 9;16(5):750. doi: 10.3390/v16050750 (PMC11126044; doi:10.3390/v16050750)

Figure supplementary S1. Sample characteristics and inclusion criteria of the analysis

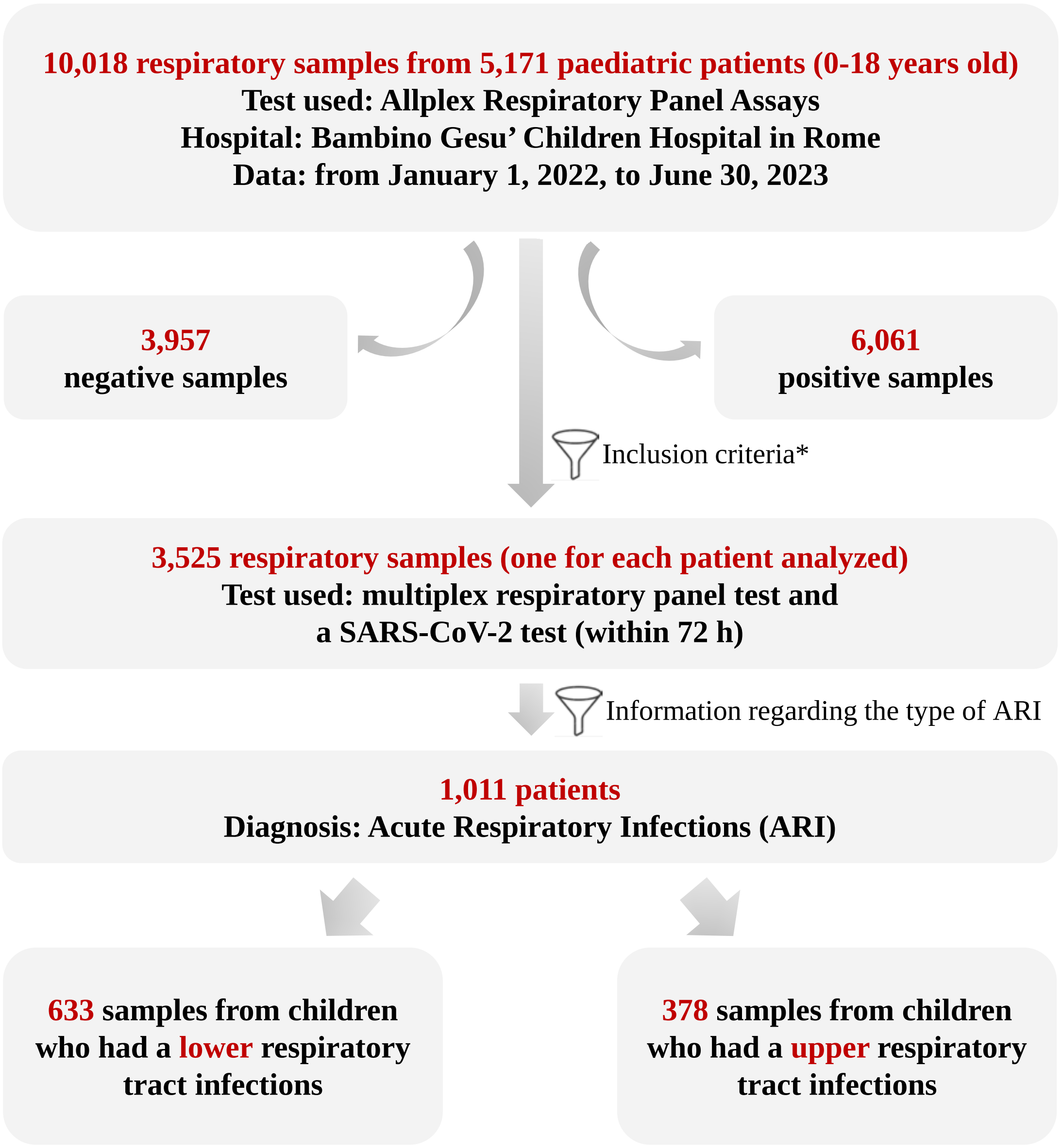

*Patients with a multiplex respiratory panel test and a SARS-CoV-2 test within 72 hours. If a patient had multiple respiratory samples, we retrospectively selected the first one that in most cases matched the one performed at the time of hospital admission (inpatient and outpatient). Respiratory specimens belonging to the same patient with a time distance >30 days were considered as a new case.

Figure supplementary S2. Distribution of community respiratory viruses from January 2022 to June 2023.

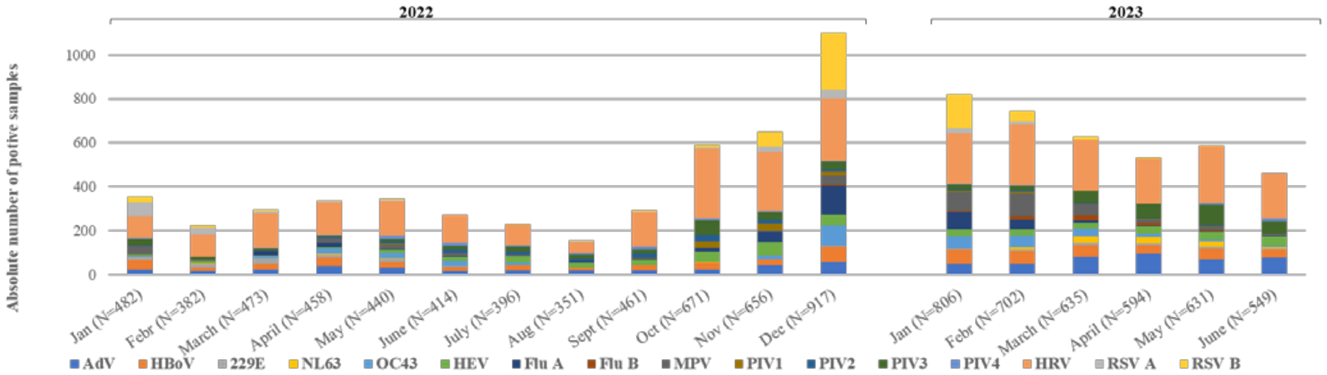

2022

	Jan (N=482)	Febr (N=382)	March (N=473)	April (N=458)	May (N=440)	June (N=414)	July (N=396)	Aug (N=351)	Sept (N=461)	Oct (N=671)	Nov (N=656)	Dec (N=917)
**AdV**	21 (4.4)	17 (4.5)	24 (5.1)	40 (8.7)	33 (7.5)	17 (4.1)	19 (4.8)	18 (5.1)	19 (4.1)	23 (3.4)	43 (6.6)	58 (6.3)
**HBoV**	43 (8.9)	17 (4.5)	25 (5.3)	39 (8.5)	26 (5.9)	20 (4.8)	23 (5.8)	14 (4)	22 (4.8)	32 (4.8)	26 (4)	69 (7.5)
**229E**	19 (3.9)	16 (4.2)	25 (5.3)	12 (2.6)	12 (2.7)	2 (0.5)	1 (0.3)	0 (0.0)	0 (0.0)	2 (0.3)	1 (0.2)	3 (0.3)
**NL63**	0 (0.0)	1 (0.3)	2 (0.4)	4 (0.9)	4 (0.9)	0 (0.0)	0 (0.0)	1 (0.3)	0 (0.0)	1 (0.1)	0 (0.0)	1 (0.1)
**OC43**	3 (0.6)	0 (0.0)	9 (1.9)	23 (5.0)	24 (5.5)	24 (5.8)	13 (3.3)	0 (0.0)	0 (0.0)	0 (0.0)	19 (2.9)	91 (9.9)
**HEV**	4 (0.8)	10 (2.6)	4 (0.8)	7 (1.5)	15 (3.4)	20 (4.8)	30 (7.6)	21 (6)	23 (5)	48 (7.2)	58 (8.8)	51 (5.6)
**Flu A**	2 (0.4)	1 (0.3)	18 (3.8)	18 (3.9)	4 (0.9)	3 (0.7)	0 (0)	8 (2.3)	6 (1.3)	15 (2.2)	45 (6.9)	131 (14.3)
**Flu B**	0 (0.0)	0 (0.0)	0 (0.0)	0 (0.0)	0 (0.0)	0 (0.0)	0 (0.0)	0 (0.0)	0 (0.0)	0 (0.0)	1 (0.2)	5 (0.5)
**MPV**	38 (7.9)	5 (1.3)	4 (0.8)	24 (5.2)	21 (4.8)	12 (2.9)	4 (1.0)	0 (0.0)	1 (0.2)	1 (0.1)	7 (1.1)	41 (4.5)
**PIV1**	0 (0.0)	0 (0.0)	0 (0.0)	0 (0.0)	1 (0.2)	0 (0.0)	1 (0.3)	0 (0.0)	4 (0.9)	30 (4.5)	32 (4.9)	19 (2.1)
**PIV2**	3 (0.6)	2 (0.5)	1 (0.2)	4 (0.9)	10 (2.3)	16 (3.9)	14 (3.5)	11 (3.1)	20 (4.3)	29 (4.3)	18 (2.7)	10 (1.1)
**PIV3**	30 (6.2)	12 (3.1)	5 (1.1)	5 (1.1)	14 (3.2)	18 (4.3)	23 (5.8)	18 (5.1)	18 (3.9)	64 (9.5)	36 (5.5)	36 (3.9)
**PIV4**	3 (0.6)	2 (0.5)	3 (0.6)	3 (0.7)	14 (3.2)	12 (2.9)	5 (1.3)	6 (1.7)	16 (3.5)	12 (1.8)	4 (0.6)	4 (0.4)
**HRV**	99 (20.5)	101 (26.4)	158 (33.4)	151 (33)	157 (35.7)	124 (30)	92 (23.2)	52 (14.8)	155 (33.6)	315 (46.9)	269 (41)	283 (30.9)
**RSV A**	65 (13.5)	28 (7.3)	11 (2.3)	5 (1.1)	7 (1.6)	2 (0.5)	1 (0.3)	5 (1.4)	4 (0.9)	5 (0.7)	23 (3.5)	40 (4.4)
**RSV B**	25 (5.2)	10 (2.6)	7 (1.5)	1 (0.2)	2 (0.5)	0 (0.0)	1 (0.3)	2 (0.6)	5 (1.1)	13 (1.9)	67 (10.2)	262 (28.6)

2023

	Jan (N=806)	Febr (N=702)	March (N=635)	April (N=594)	May (N=631)	June (N=549)
**AdV**	48 (6.0)	48 (6.8)	82 (12.9)	94 (15.8)	69 (10.9)	79 (14.4)
**HBoV**	67 (8.3)	61 (8.7)	53 (8.3)	40 (6.7)	49 (7.8)	37 (6.7)
**229E**	2 (0.2)	5 (0.7)	10 (1.6)	7 (1.2)	9 (1.4)	3 (0.5)
**NL63**	2 (0.2)	12 (1.7)	30 (4.7)	31 (5.2)	24 (3.8)	5 (0.9)
**OC43**	56 (6.9)	50 (7.1)	35 (5.5)	16 (2.7)	9 (1.4)	8 (1.5)
**HEV**	32 (4)	30 (4.3)	27 (4.3)	33 (5.6)	33 (5.2)	43 (7.8)
**Flu A**	77 (9.6)	43 (6.1)	10 (1.6)	3 (0.5)	4 (0.6)	1 (0.2)
**Flu B**	6 (0.7)	16 (2.3)	23 (3.6)	13 (2.2)	7 (1.1)	1 (0.2)
**MPV**	85 (10.5)	105 (15)	53 (8.3)	13 (2.2)	14 (2.2)	10 (1.8)
**PIV1**	6 (0.7)	9 (1.3)	0 (0.0)	3 (0.5)	3 (0.5)	0 (0.0)
**PIV2**	5 (0.6)	6 (0.9)	1 (0.2)	4 (0.7)	6 (1.0)	1 (0.2)
**PIV3**	23 (2.9)	20 (2.8)	56 (8.8)	65 (10.9)	93 (14.7)	56 (10.2)
**PIV4**	4 (0.5)	1 (0.1)	0 (0.0)	1 (0.2)	5 (0.8)	12 (2.2)
**HRV**	231 (28.7)	277 (39.5)	233 (36.7)	204 (34.3)	259 (41)	204 (37.2)
**RSV A**	24 (3.0)	14 (2.0)	3 (0.5)	0 (0.0)	0 (0.0)	2 (0.4)
**RSV B**	154 (19.1)	49 (7.0)	10 (1.6)	6 (1.0)	3 (0.5)	0 (0.0)

ADV: Adenovirus, HBoV: Human bocavirus, HCoV-229E: Human coronavirus 229E, HCoV-OC43: Human coronavirus OC43, HeV: Human enterovirus, FluA: Influenza A virus, FluB: Influenza B virus; MPV: Human metapneumovirus, PIV1: Human parainfluenza virus 1, PIV2: Human parainfluenza virus 2, PIV3: Human parainfluenza virus 3, PIV4: Human parainfluenza virus 4, HRV: Human rhinovirus, RSVA: Respiratory syncytial virus A, RSVB: Respiratory syncytial virus B, SARS-CoV-2: Severe acute respiratory syndrome coronavirus.

Figure supplementary S3. Distribution and prevalence of viral mono and co-detection within age groups

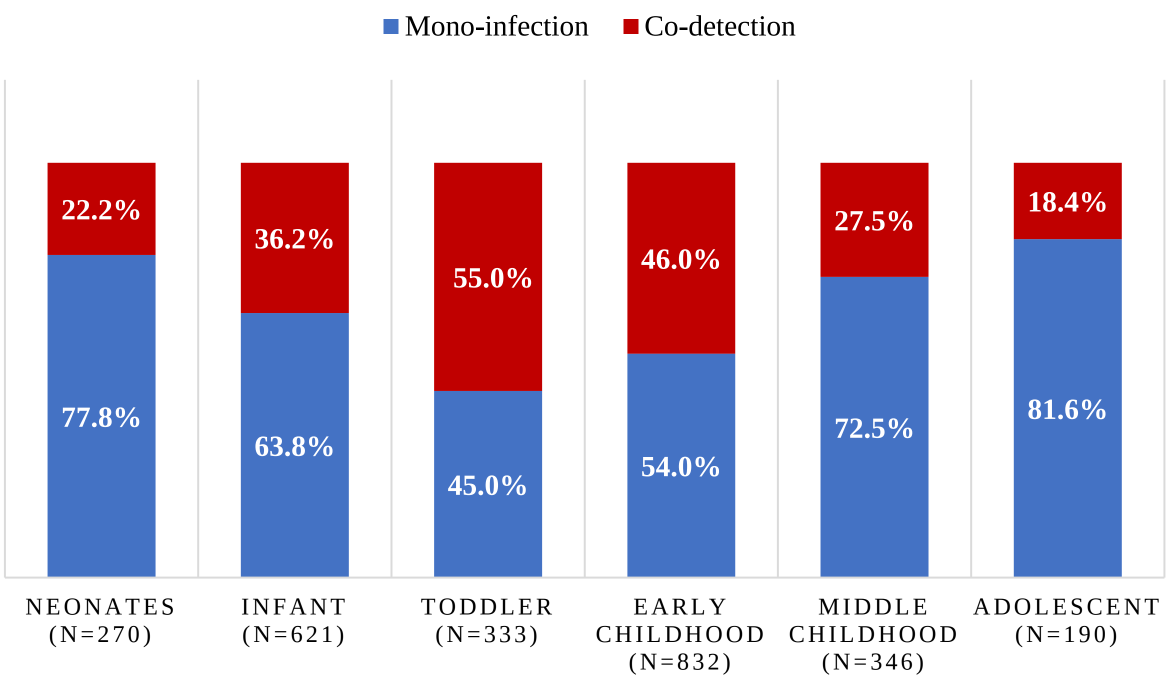

Supplement: Supplementary file 1 [file viruses-16-00750-s001.zip › Supplementary_Figures S1-S3.pdf]
